# The Role of Non-Catalytic Domains of Hrp3 in Nucleosome Remodeling

**DOI:** 10.3390/ijms22041793

**Published:** 2021-02-11

**Authors:** Wenbo Dong, Punit Prasad, Andreas Lennartsson, Karl Ekwall

**Affiliations:** 1 Department of Biosciences and Nutrition, Karolinska Institutet NEO, 141 83 Huddinge, Sweden; wenbo.dong@brbiotech.com (W.D.); andreas.lennartsson@ki.se (A.L.); 2 Cancer Biology Group, Institute of Life Sciences, NALCO Square, Bhubaneswar, Odhisa 751023, India

**Keywords:** S*. pombe* nucleosomes, Hrp3, chromatin remodeling, ATPase activity

## Abstract

The Helicase-related protein 3 (Hrp3), an ATP-dependent chromatin remodeling enzyme from the CHD family, is crucial for maintaining global nucleosome occupancy in *Schizosaccharomyces pombe* (*S. pombe)*. Although the ATPase domain of Hrp3 is essential for chromatin remodeling, the contribution of non-ATPase domains of Hrp3 is still unclear. Here, we investigated the role of non-ATPase domains using in vitro methods. In our study, we expressed and purified recombinant *S. pombe* histone proteins, reconstituted them into histone octamers, and assembled nucleosome core particles. Using reconstituted nucleosomes and affinity-purified wild type and mutant Hrp3 from *S. pombe* we created a homogeneous in vitro system to evaluate the ATP hydrolyzing capacity of truncated Hrp3 proteins. We found that all non-ATPase domain deletions (∆chromo, ∆SANT, ∆SLIDE, and ∆coupling region) lead to reduced ATP hydrolyzing activities in vitro with DNA or nucleosome substrates. Only the coupling region deletion showed moderate stimulation of ATPase activity with the nucleosome. Interestingly, affinity-purified Hrp3 showed co-purification with all core histones suggesting a strong association with the nucleosomes in vivo. However, affinity-purified Hrp3 mutant with SANT and coupling regions deletion showed complete loss of interactions with the nucleosomes, while SLIDE and chromodomain deletions reduced Hrp3 interactions with the nucleosomes. Taken together, nucleosome association and ATPase stimulation by DNA or nucleosomes substrate suggest that the enzymatic activity of Hrp3 is fine-tuned by unique contributions of all four non-catalytic domains.

## 1. Introduction

Chromatin is the fundamental molecular structure for packing DNA into chromosomes. In eukaryotic cells, DNA is wrapped around histone octamers, which consist of two molecules of each histone H2A, H2B, H3, and H4 [1]. Chromatin remodelers are a group of enzymes that can utilize the energy of hydrolyzing ATP to modulate chromatin structures through diverse mechanisms, which include nucleosome sliding and repositioning, nucleosome unwrapping, histone eviction, and histone variant exchange [2,3]. Most of the chromatin remodelers share a conserved ATPase domain, and categorized into several families according to the presence or absence of accessory domains, for instance the SWI/SNF, ISWI, CHD, and INO80 families. Among them, the Chromo helicase domain (CHD) family is characterized by the presence of a pair of chromo-domains present in the N-terminal part of the protein. Chromo-domains of CHD have been shown to recognize histone H3 methylated at lysine 4 (H3K4me) [4]. CHD proteins identified in all eukaryotic organisms and play crucial roles in different aspects of cellular processes. In humans, CHD proteins are further classified into different subgroups: CHD1–2, Mi2/NURD (CHD3-4), and CHD5–9 [5]. The human CHD1 enzyme binds to H3K4me3 through its paired chromo-domains [6]. It is required for maintaining an open chromatin state at active genes [7]. Inactivation or dysfunction of CHD1 has been found in several cancers, including breast cancer and prostate cancer [8,9]. Recently, a role for CHD1 in learning and memory functions was discovered in a mouse model [10]. Mutations in its homolog CHD2 has also been found to be associated with human cancers, such as chronic lymphoblastic leukemia (CLL) [11]. Both CHD1 and CHD2 have been shown to regulate histone H3/H3.3 occupancy and chromatin accessibility at the transcription start site (TSS) region of their target genes [12]. In *Saccharomyces cerevisiae (S. cerevisiae)*, ScCHD1 is the only known CHD orthologue [2]. ScCHD1 regulates the chromatin structure through nucleosome spacing or sliding and nucleosome assembly [2,13,14]. It can also affect H3 turnover and deposition at 5′ ends and 3′ends genes [15]. The chromo-domains of ScCHD1 block its capacity of substrate stimulation by DNA, which results in its preference for nucleosome as a substrate [16,17]. The SANT and SLIDE domain constitute the DNA binding region of ScCHD1 [18]. A small region between the ATPase and the SANT domains was shown to be important for the nucleosome spacing activity of ScCHD1 but not for nucleosome assembly [19]. According to its function, this region was named the coupling region. The identification of the coupling region suggested that the remodeling process of ScCHD1 is divided into two distinct steps, nucleosome assembly, and nucleosome spacing. These two steps are sequentially connected and both steps are ATP dependent [17]. The Cryo-EM (electron microscopy) structure of ScCHD1 together with the nucleosome revealed molecular details of the remodeling mechanism including contacts of SANT and SLIDE domains with DNA [20]. These findings suggest that a key step in remodeling occurs when the chromo-domains are in contact with DNA allowing for a specific lobe in the ATPase domain to interact with histone H4, and this contact is crucial for the following ATPase dependent DNA translocation step.

The model organism fission yeast (*S. pombe*), has a small genome size. The chromatin organization and gene regulation processes in fission yeast are similar to those of human cells [21]. Fission yeast has two CHD1 homologs, Hrp1 and Hrp3. The two homologs have overlapping functions in the regulation of nucleosome spacing and the prevention of cryptic transcription [15,22]. However, Hrp3 has a specific role in nucleosome disassembly at some loci such as the *fpb1+* gene [23]. In this study, we aimed to characterize the functions of all non-ATPase domains of Hrp3 in *S. pombe*. To this end, we engineered *S. pombe* strains carrying internal domain deletions of Hrp3 and a tandem affinity purification (TAP) epitope-tag at its C-terminus (∆chromo, ∆SANT, ∆SLIDE, and ∆coupling region). We affinity purified wild type (WT) and mutant Hrp3 proteins from *S. pombe* and used them for in vitro assays. Our group reported earlier that Hrp3 co-purifies specifically with all four core histones from *S. pombe* [22]. Here we found that the mutant forms of Hrp3 all showed reduced or abolished interactions with the nucleosomes in comparison with WT Hrp3. To study Hrp3 remodeling in vitro we expressed and purified recombinant *S. pombe* histones, made octamers, and successfully reconstituted nucleosomes. Using this homogenous in vitro system with fission yeast components, we then analyzed the ATP hydrolyzing capacity of purified WT and mutant forms of Hrp3. We found that all of the Hrp3 mutants showed a compromised ATPase activity consistent with the observed weakened nucleosome interactions. Only the coupling region deletion showed a moderate stimulation of ATPase activity with the nucleosome. Thus, our results suggest that SANT, SLIDE, and chromo-domains of Hrp3 contribute to both ATPase activity and nucleosome interactions in a non-redundant way. Further experiments are required to understand the precise role of each domain in chromatin remodeling.

## 2. Results

### 2.1. Purification of Fission Yeast Recombinant Histones and Reconstitution of Mononucleosomes

**Figure 1:**
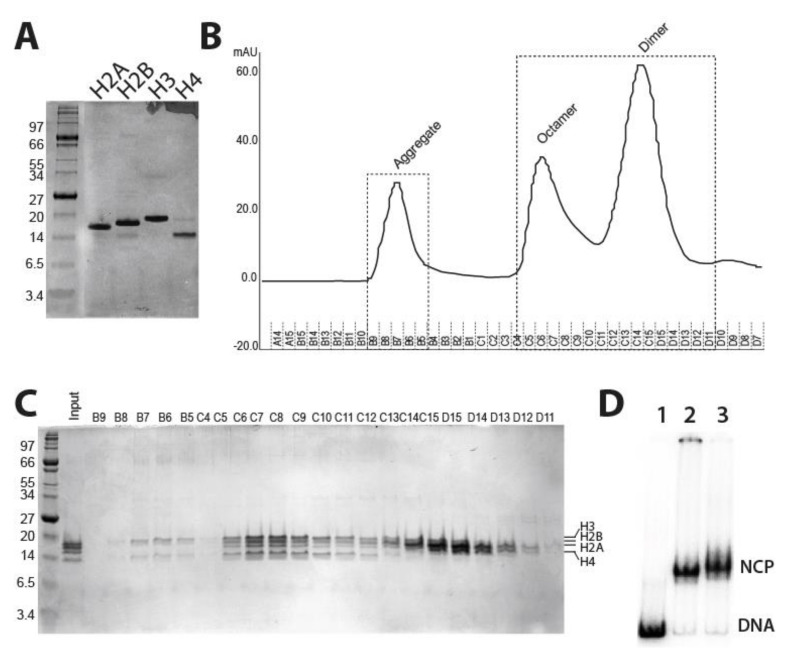
Purification of histone proteins, reconstitution of the histone octamer, and nucleosome core particles preparation: (**A**) Purified recombinant *S. pombe* histone proteins are separated on the denaturing 18% SDS-PAGE gel. (**B**) Refolded histone octamers were purified using the Superdex-200 gel exclusion column. The three peaks of aggregates, octamer, and dimers are shown. (**C**) Eluted protein fractions corresponding to three different peaks were separated on SDS-PAGE, Coomassie stained, quantified, and pooled (bottom panel). (**D**) Nucleosome core particle (NCP) on 601 PCR amplified DNA template having 70 bp of linker DNA (217 bp total length) with *Xenopus* histone (lane 2) and fission yeast octamer (lane 3), respectively, was reconstituted using salt dilution method and separated on 4% native PAGE gel.

The small genome size and a chromatin organization similar to higher eukaryotes make fission yeast an ideal model for studies of nucleosome remodeling. Therefore, it is imperative to purify fission yeast nucleosomes for biochemistry experiments. Towards this, the four core histone genes were amplified from *S. pombe* genomic DNA and cloned in pET-13 vector for overexpression in *E. coli* BL21 strains (Figure S1). To overexpress recombinant *S. pombe* histones, we tested different *E.coli* strains in order to optimize *S. pombe* histone gene expression to achieve the highest expression levels such that the proteins aggregate into inclusion bodies (Figure S1). Recombinant *S. pombe* histone spH2A, spH2B, and spH3 proteins overexpression were optimized in BL21 DE3 pLys (Stratagene), while BL21 Star™ (DE3) cells were used for histone spH4 overexpression. BL21 Star™ (DE3) cells (Invitrogen) have higher mRNA stability and low background for uninduced proteins, which resulted in stable and higher protein expression levels (Figure S1). Overexpressed histones in *E. coli* formed inclusion bodies. Next, we extracted *S. pombe* histones from unfolded inclusion bodies that were subsequently purified by gel filtration chromatography, followed by ion-exchange chromatography using HPLC (Figure 1A).

To reconstitute and refold histones into octamers, the molar ratio of 1.1:1.1:1:1 of H2A:H2B:H3:H4 were mixed and dialyzed in a refolding buffer. An excess of spH2A and spH2B was used to avoid the formation of histone hexamers (H2A/H2B dimer + H3/H4 tetramer). The refolded histones were concentrated and octamers were purified using the Superdex-S200 gel exclusion column (Figure 1B). The eluted peak fractions were checked using a denaturing PAGE gel and densitometry quantification of each histone band was done to obtain peak fractions that can be pooled as octamers. The aggregate peaks B9 to C5 showed poor histone levels, which aggregate, along with the residual DNA. Histone peak fractions from C15 to D12 showed spH2A and spH2B dimer peaks. The aggregate and dimer peak fractions were eliminated and four fractions C–C9 with equal histone protein levels were pooled, concentrated, and mixed with 100% glycerol to 10 mg/mL final concentration for long term storage in the freezer (Figure 1C). To check the ability of octamers to form nucleosomes, we used 217 bp of PCR amplified DNA, consisting of 147 bp of 601 nucleosome positioning sequence and 70 bp of linker DNA at one end. Nucleosomes were reconstituted using the salt dilution method [22] by mixing equimolar ratios of octamers and DNA. Reconstituted 70N0 nucleosomes were checked on 4% native PAGE gels, which shows a distinct shift in electrophoretic mobility of reconstituted nucleosomes compared with mock DNA (Figure 1D). As a control, Xenopus histone octamer was used to make the nucleosomes along with fission yeast octamer. There is a moderate supershift in the mobility of the fission nucleosomes suggesting that the nucleosome core particles (NCP) are not as compact as for *Xenopus* nucleosomes. In sum, the fission yeast nucleosomes had been successfully expressed, purified, and reconstituted into mononucleosomes.

### 2.2. Generation of Hrp3 Mutants and Affinity Purification of WT and Mutant Proteins from Fission Yeast

**Figure 2:**
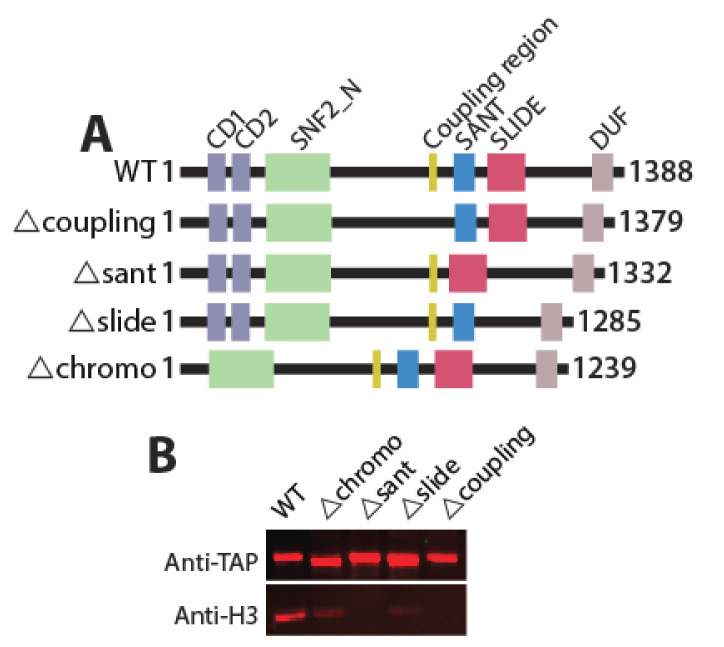
Schema of Hrp3 domain organization, their deletions to generate mutant Hrp3, and affinity purification of WT/mutant Hrp3 protein. (**A**) A schematic representation of Hrp3 with all domains (WT) and internal domain deletion (mutants). The change in the protein length of Hrp3 upon domain deletion is shown. Strains harboring these constructs are Hu2402 (WT), Hu3060 (∆chromo), Hu3061 (∆sant), Hu3062 (∆slide) Hu3063 (∆coupling) with complete genotypes indicated in Table 1. (**B**) Western blot showing the affinity-purified wild type and domain deleted Hrp3 proteins and co-purified histone H3.

Chromatin remodeling complexes have domains other than catalytic domain to assist in regulating nucleosome remodeling, interactions with the nucleosomes and DNA. Together, these domains contribute to chromatin recognition and nucleosome remodeling. Loss of any of these accessory domains may result in abrogation of ATP hydrolysis, DNA binding, nucleosome interaction, or nucleosome remodeling [18]. Hrp3 has tandem chromodomains, a SNF2 helicase domain, a coupling region, SANT, SLIDE domains, and a domain of the unknown function (DUF) (Figure 2A). Together these domains are likely to contribute to efficient chromatin remodeling. Chromodomains are known to interact with the histones, SANT/SLIDE with the DNA and coupling region, a stretch of amino acids between the helicase domain and DNA binding domain essential for chromatin remodeling [19,24]. To explore the functional significance of different non-ATPase domains of Hrp3, we generated strains with internal deletions of chromo-domain (amino acids 192–340), SANT domain (amino acids 994–1049), SLIDE domain (amino acids 1103–1205), and coupling region (amino acids 927–935) (Figure 2A). Our domain deletion strategy is shown in Figure S2.

We constructed wild type or mutant Hrp3 proteins with a TAP epitope-tag at the C-terminus for affinity purification of the native proteins. We used 10–12 L of *S. pombe* cell cultures (See Materials and Methods). The resulting cell lysates were bound with IgG beads and eluted using TEV protease. Eluted proteins were quantified using BSA standards in SDS-PAGE and equimolar amounts of protein were separated on SDS-PAGE and stained with Sypro ruby. The mutant Hrp3 proteins have different mobility on the denaturing gel due to their domain deletions (Figure S2B). Western blots using anti-TAP antibody show a moderate change in the mobility of the proteins upon internal deletion. (Figure 2B, upper panel). We have earlier reported the association of Hrp3 with the nucleosomes as all four histones co-purify with Hrp3 [22]. Therefore, we now focused on mutant Hrp3 enzymes and their association with the nucleosomes. To further explore the effect of domain deletion on Hrp3 nucleosome interaction in vivo, we also performed an anti-H3 western blot using purified WT and mutant Hrp3 (Figure 2B; bottom panel). Interestingly, we found that the association of mutant Hrp3 with the nucleosomes were severely compromised. Deletion of the SANT domain and coupling region resulted in complete loss of Hrp3-nucleosomal interaction while chromo and SLIDE domains showed much weaker interaction with the nucleosomes (Figure 2B; lower panel). This analysis revealed that all the Hrp3 deletions showed a significant reduction in the binding of histone H3 in comparison to WT Hrp3.

### 2.3. ATPase Activity of WT and Mutant Hrp3 with DNA and Nucleosomal Substrates

**Figure 3:**
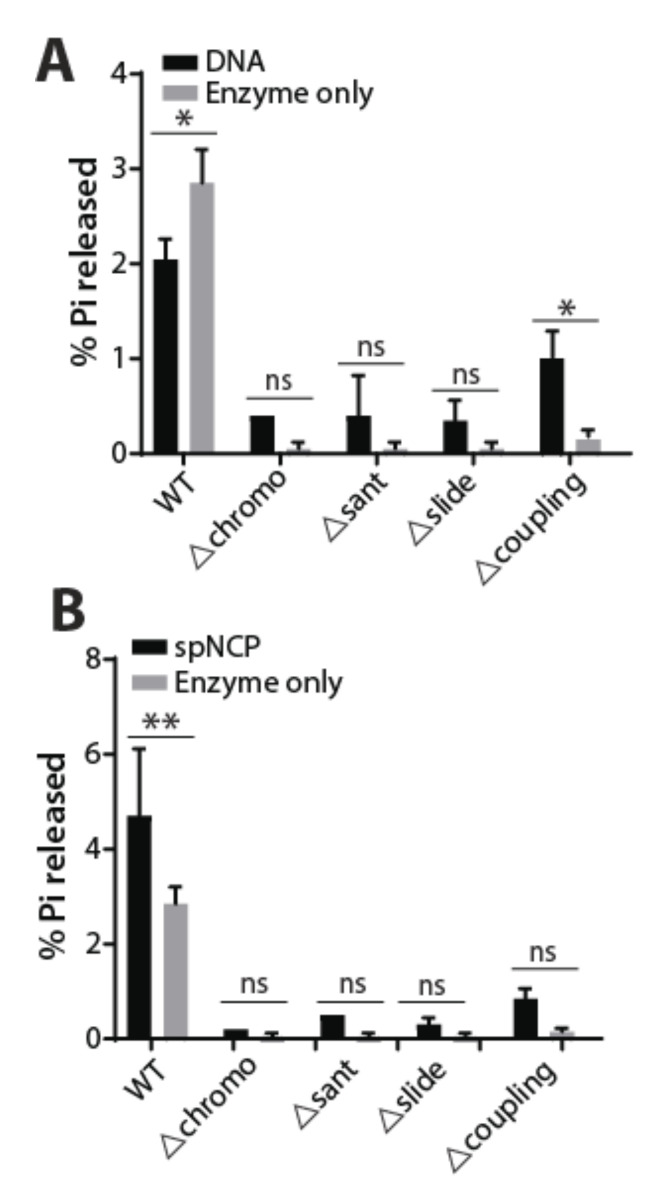
ATPase activities for wild type and Hrp3 mutants with DNA and nucleosome substrates: WT or mutant Hrp3 (30 femto moles) were used for ATP hydrolysis with DNA (**A**) or *S. pombe* nucleosomes (**B**). The *y*-axis shows the percentage of inorganic phosphate (Pi) released upon ATP hydrolysis by WT or mutant Hrp3. Graphpad Prism was used to plot the graphs and to calculate the significance of the catalytic activity using 2way ANOVA. For comparison between DNA-enzyme alone for WT and ∆coupling region are 0.018 and 0.0118, respectively, while for spNCP-enzyme alone the significance value was 0.0068. The significance was calculated from two biological replicates. Other comparisons did not give significance and are marked as ‘ns’. The significance is indicated in the figure (* *p* < 0.05; ** *p* < 0.01).

Next, we wanted to assess the catalytic function of the WT and mutant Hrp3. Therefore, we tested their ability to hydrolyze ATP using DNA and 70N0 end-positioned nucleosomes as their substrates. We titrated different amounts of WT or mutant Hrp3 with DNA, nucleosomes, and enzyme alone (no substrate) and tested their extent of ATP hydrolysis by calculating the amount of free radio-labeled inorganic phosphate (Pi) using thin-layer chromatography (Figure S3). In general, ATP-hydrolysis of wild type Hrp3 was significantly higher compared with mutant Hrp3 proteins at various concentrations (Figure 3A,B and Figure S3). Moreover, WT Hrp3 showed higher ATPase activity with the nucleosome than DNA, which was consistent with an earlier report (compare Figure 3A,B) [16]. Among Hrp3 mutants, only the coupling region deletion showed higher ATPase activity (Figure S3). This observation is in line with two reports on the ScCHD1 coupling region deletion [19,24]. Interestingly, the WT Hrp3 enzyme control showed concentration-dependent ATP hydrolysis activity in the absence of substrates suggesting that the co-purified nucleosomes along with Hrp3 are being used as the substrates (Figure 2B and Figure S3A). Contrary to this, all four mutant Hrp3 proteins showed significantly reduced ATPase activity and their interactions with the nucleosomes were significantly compromised (Figure S3). Thus, loss of accessory domains of Hrp3 other than the ATPase domain abrogates ATPase stimulation by the substrates.

## 3. Discussion

In this study, we describe the successful purification and reconstitution of *S. pombe* nucleosomes in vitro for biochemical characterization of Hrp3. The methodology for spNCP assembly will likely be useful for other studies in fission yeast since it provides a homogenous system in a popular experimental model organism for studies of chromatin states [25]. Using reconstituted *S. pombe* nucleosomes, we used CHD1 homolog Hrp3 to show the efficacy of ATP hydrolyzing ability of Hrp3. We found that nucleosome-stimulated ATPase activity of Hrp3 was higher compared with DNA substrates supporting the fact that CHD1 has higher ATPase activity with the nucleosomal substrates. Next, we wanted to understand the role of accessory domains of Hrp3 in ATPase activity. Therefore, we created internal deletions of chromo, coupling region, SANT, and SLIDE domains and affinity-purified them for characterization. We found that the loss of accessory domains significantly compromises the ATP stimulation either with DNA or nucleosomal substrates. In our previous study, we observed that affinity purification of Hrp3 but not Hrp1 co-purifies with all the histones showing that Hrp3 strongly interacts with the nucleosomes in vivo [22]. Here, we observed that the affinity purification of mutant Hrp3 either weakens or result in a complete loss of Hrp3-nucleosome interaction. This loss of interaction is potentially the reason for significantly reduced ATPase activity of the mutant Hrp3.

Recombinant histones from various eukaryotes have been purified to-date despite being high levels of identity in different histones. Among histones, H3 and H4 are highly conserved across species while H2A and H2B show less conservation. Although we were the first to report the purification and reconstitution of *S. pombe* into nucleosomal arrays, we did not explicitly explain the histone purification and optimization process [22]. Koyama et al., reported purification of *S. pombe* recombinant His-tagged histones and reconstituted them into nucleosomes [26]. They found that the stability of the *S. pombe* nucleosome was reduced compared to the human nucleosome. Our study further elaborates on the purification of untagged recombinant histones using traditional histone purification as described by Luger et al. [27] and mononucleosome reconstitution. The functionality of the assembled nucleosomes was previously studied for spacing assays [22] and here for ATPase activity of Hrp3 using mononucleosomes.

*S. pombe* has two CHD1 homologs Hrp1 and Hrp3, with overlapping functions in nucleosome spacing and prevention of cryptic anti-sense transcription, and Hrp3 has a higher affinity for nucleosomes compared to Hrp1 [22]. Micrococcal nuclease digestion of chromatin followed by DNA hybridization using tiling arrays showed that the absence of Hrp3 but not Hrp1 significantly affects nucleosome spacing indicating Hrp3 as a key chromatin remodeling enzyme for nucleosome spacing. Protein alignment with Hrp3 homologs showed conserved accessory chromo, SANT, SLIDE, and coupling region domains [18,19]. Here we focused on the role of the non-catalytic (non-ATPase) domains of Hrp3 and we made four different domain deletions of chromo-domains, SANT, SLIDE, and the coupling region. Our results suggest that these non-ATPase domains contribute to chromatin interaction and ATP hydrolysis. The loss of chromodomain in *S. cerevisiae* CHD1 results in enhanced ATPase activity with both nucleosome and DNA substrates [16,18], contrary to Hrp3 where ATPase activity is significantly compromised. DNA binding domains, SANT and SLIDE, however, are essential for efficient ATPase activity for both CHD1 and Hrp3 [16]. These DNA binding domains are essential for directional sliding of the nucleosomes by orienting themselves towards extranucleosomal DNA [28,29]. The coupling region is a short stretch of amino acids (~10–30 aa), between ATPase domain and DNA binding domain is conserved in CHD1 across different eukaryotes [19]. *S. pombe* Hrp3 coupling region of around 8 amino acids are highly conserved across different eukaryotic CHD1 and deletion of this region reduces the overall ATPase activity although it is still somewhat stimulated by additions of nucleosomes or DNA (Figure 3 and Figure S3). Although *S. cerevisiae* CHD1 (∆932–949 aa) showed more nucleosomal ATPase activity compared with the DNA substrate [19], we found similar levels of ATPase stimulation with both the substrates (Figure S3E). It is interesting that the coupling region deletion is the only mutant construct in this study that responds to additions of DNA or nucleosomes. This may suggest a unique role for this region in the nucleosome remodeling mechanism. In general all domain deletions we made of Hrp3 abrogated the strong association of nucleosomes with Hrp3 (Figure 2B). Likely as a consequence of the weakened nucleosome interaction we observed that the ATPase activity of Hrp3 deletions was also generally low in vitro. The addition of spNCP substrate failed to give a significant stimulation of ATPase activity of the mutant enzymes. This suggests that a strong nucleosome interaction is of key importance for Hrp3 activity.

Recently, the cryo-EM structure of *S. cerevisiae* CHD1 enzyme (ScCHD1) in contact with nucleosome was determined [20]. This pioneering work showed that the DNA binding capacity of SANT, SLIDE, and chromo-domains have distinct roles in positioning specific parts of the central ATPase domain in contact with histone H4 to allow for the ATP-dependent DNA translocation step. In light of this study, it seems logical that SANT, SLIDE, and chromo-domains would have unique roles required for the enzymatic activity of Hrp3. Further mechanistic studies are required to determine the precise role of each non-ATPase domain in nucleosome remodeling by Hrp3. Our finding that Hrp3 SANT deletion showed an increased preference for DNA as a substrate at higher enzyme concentrations may suggest that the SANT and SLIDE domains affect the DNA binding properties of Hrp3 in different ways.

## 4. Materials and Methods

### 4.1. Fission Yeast Strains

The genotypes of the *S.pombe* strains used in this study are listed in Table 1. Yeast cells were cultured in standard media containing 5.0 g/L of yeast extract supplemented with 225 mg/L adenine, histidine, leucine, uracil, and lysine hydrochloride, 2% glucose) (reference: Forsburg Lab https://dornsife.usc.edu/pombenet/ (accessed on 11 February 2021). The generation of domain deleted *S. pombe* strains is described in Figure S2.

### 4.2. Expression of Histone Proteins

The *S. pombe* histones spH2A, spH2B, and spH3 cDNAs were cloned into pET-13 plasmids, and the transformation was done in *E.coli strains* BL21 (DE3) pLyS (Stratagene). The transformation for spH4 plasmid was done in *E.coli* strains BL21 star (DE3) cells (Invitrogen). Transformed colonies were selected based on Kanamycin resistance. Individual bacterial colonies containing the respective histone gene plasmids were, inoculated in 3 mL LB media having 34 mg/mL Kanamycin and 34 mg/mL chloramphenicol and incubate at 37 °C until OD = 0.3 to 0.4. For histone H4, colonies were inoculated in 10 mL LB media + 12.5 mM MgCl_2_ and MgSO_4_, incubated at 37 °C until OD = 0.6. Bacterial cells were induced with 0.5 mM IPTG and incubated for 2.5 to 3 h at 37 °C. 1 mL of each culture was harvested, cells were re-suspended in loading buffer and sonicated. The samples were heated at 95 °C for 2 min and run in 18% SDS-PAGE gels to check histone expression and to select the best-expressing colonies for large-scale induction. Large cultures were inoculated in 12 L for spH4 and 6 lit for spH2A, spH2B, and spH3) from the pre-culture of the chosen colonies. Media compositions and IPTG induction were the same as for the mini-expression cultures. The cells are harvested and stored at −80 °C for protein extraction.

### 4.3. Preparation of Inclusion Bodies

Bacterial cell pellets were thawed and re-suspended in wash buffer (50 mM Tris-Cl pH 7.5, 100 mM NaCl, 1 mM EDTA, 5 mM BME, and 0.2 mM PMSF), and transferred to ice immediately after thawing. The cells were lysed and sonicated using a microprobe sonicator at 40% output with 10 s pulses and 10 s pause for a total of 2 min or until the viscosity of the suspended cells changes to water-like consistency. The cells were kept on ice during the whole process. The cells were transferred into a pre-chilled centrifuge bottle and inclusion bodies were centrifuged at 10,000 rpm at 4 °C. Inclusion bodies pellet is washed twice with wash buffer (50 mM Tris-Cl pH 7.5, 100 mM NaCl, 1 mM EDTA, 5 mM BME and 0.2 mM PMSF + 1% Triton X-100).

### 4.4. Purification of Fission Yeast Histone Proteins

Histone proteins were purified from inclusion bodies through gel filtration. Pre-washed Sephacryl-S200 column was equilibrated with Urea buffer (7 M Urea, 1 M NaCl, 20 mM NaAc pH5.2, 1 mM NaEDTA and 5 mM BME). The inclusion bodies from 6 L bacterial culture for all histones except spH4 were homogenized by adding DMSO 400 μL DMSO. For spH4, inclusion bodies from 2 L bacterial culture were suspended in 400 μL DMSO. The sample was continuously mixed at RT for half an hour, then 5–8 mL of unfolding buffer was added (7 M Guanidine hydrochloride, 20 mM Tris-Cl pH7.5, 10 Mm DTT). The samples were centrifuged at 20,000× *g* for 10 min at RT, the supernatant was collected and loaded onto an equilibrated column. To run the column, at least two column volumes of urea buffer was required. Protein fractions were collected till absorbance λ = 280 nm was reduced. Fractions with peaks were analyzed by SDS-PAGE as discussed above. All the fractions for each histone were pooled together and dialyzed in at least 2 L water containing 5 mM BME by using a 6–8 KDa cut-off dialysis bag or tube. The dialysis buffer was changed three times: at 2, 4, and 12–16 h (overnight). The proteins were snap-frozen in liquid nitrogen and lyophilized. The second round of histone purification was carried out using ion-exchange chromatography. Preparations of spH3 or spH4 were dissolved in 1–2 mL SAU-200 buffer (200 mM salt) and purify through Source15S column with buffer containing 7 M Urea, 20 mM NaAc, 5 mM BME, 1 mM EDTA with a salt gradient from 200 mM to 1000 mM. For spH2A or spH2B, the histones were dissolved in 1–2 mL SAU-100 buffer (100 mM) and purified through Source 15S columns with a salt gradient from 100 mM to 1000 mM. Fractions were collected and analyzed by SDS-PAGE. The dialysis, freezing, and lyophilize steps were the same as previously described [30].

### 4.5. Histone Octamer Refolding and Purification

Individual lyophilized histone proteins were dissolved in an unfolding buffer and kept at RT for 1 h. The histones were quantified on 18% SDS-PAGE using BSA (Pierce) standards and were mixed in the ratio of 1.1:1.1:1:1 for spH2A, spH2B, spH3, spH4, respectively. The histone mixture was dialyzed in 2 L of refolding buffer (2 M NaCl, 20 mM Tris-Cl pH 5.2, 1 mM BME), with three changes at 2, 4, and 12–16 h (overnight), respectively. The dialyzed mixture was concentrated to less using Amicon^®^ ultra centrifugal filters (Millipore) with a molecular weight cutoff of 3 KDa to approximately 2 mL and loaded on to the Superdex S200, which is pre-calibrated with 2–3 column volumes of the refolding buffer. Alternate fractions for each peak were collected and checked on 18% SDS-PAGE to determine the ratios for each of the histones by densitometry analysis post staining SDS-PAGE gels. The fractions having equimolar ratios of all histones were pooled, concentrated, and mixed with glycerol (50% final concentration) and stored at −20 °C [30,31].

### 4.6. Nucleosome Reconstitution

End-positioned nucleosomes, 70N0 DNA, having 601 nucleosome positioning sequence of 147 bp with 70 bp of extranucleosomal DNA on one side was PCR amplified from the p199-1 plasmid. The probe DNA by PCR in a similar manner with 5′ end-labeling of the forward primer by γP-32. The equimolar ratio of PCR amplified DNA (carrier DNA along with the probe) and WT histone octamer preparation was mixed in the 1X reaction buffer containing 25 mM Tris-Cl pH 8.0, 1 mM BME, 0.05% NP40, 0.5 mg/mL BSA, 1.5 M NaCl, 0.5 mg/mL BSA, 0.35× TE buffer. The initial sodium salt concentration was 1910 mM. The reaction mix was incubated at 30 °C for 30 min. Next, the NaCl concentration was reduced stepwise to a final concentration of 1010, 750, 500, 300, and 100 mM. The reaction mix was incubated at 30 °C for 45 min for each salt dilution step. The nucleosome reconstitution was checked by loading 3 μL of the nucleosome preparation on native PAGE gels in 0.5× TBE buffer run at 80 volts for an hour. The gel was subjected to autoradiography [30].

### 4.7. Affinity-Purification of Wild Type and Mutant Hrp3 Proteins

TAP-tagged wild type and mutant Hrp3 strains were grown to log-phase at 30 °C. The cells were harvested and ground for at least 6 cycles using a Freezer/Mill 6850 (SPEX CertiPrep). The resulting yeast powder was mixed with 3X buffer A (200 mM K-Hepes, pH 7.8, 15 mM KCl, 1.5 mM MgCl2, 0.5 mM EDTA, 15% glycerol, 1 mM DTT, and protease inhibitor cocktail), and diluted to 1×. The ground yeast powder was mixed with the buffer for 2–3 h in the cold room until all the clumps were uniformly suspended. The yeast debris was spun down and the supernatant was collected. 1/9 volume 2 M KCl was added and the liquid lysate was ultra-centrifuged at 42,000 rpm, 30 min, at 4 °C, and the supernatant was collected without disturbing the pellet. The cell extracts were incubated with IgG beads (150 uL beads is needed for 40 mL extracts) that were pre-equilibrated by IgG buffer (10 mM Tris-HCl pH 8.0, 150 mM NaCl, 10% glycerol, 1x proteinase inhibitor cocktail, 0.5 mM PMSF, 0.4 mM DTT). The mixtures were rotated at 4 °C for 1 h, ~20rpm. The beads were spun down and washed in the following order: 5 times of 10× beads volume IgG buffer, twice of 10× beads volume wash buffer (10 mM Tris-HCl, 300 mM NaCl, 0.5 mM EDTA, 0.05% NP-40, 10% Glycerol, 0.4 mM DTT + PI), 3 times of 10× beads volume TEV buffer (10 mM Tris-HCl, 150 mM NaCl, 0.5 mM EDTA, 0.05% NP-40, 10% Glycerol, pH 7.6–8 adjusted with HCl, 0.4 mM DTT + PI), twice of 10x beads volume TEV buffer (1mM DTT, no PI). Proteins were cleaved with TEV protease and eluted from the beads by incubating at 200 U enzyme for 1 h, with rotation at 16 °C.

### 4.8. In Vitro ATPase Activity Assay

Reconstituted nucleosomes were used as a substrate for purified WT Hrp3 or the different Hrp3 domain deletions to assess their ATP hydrolysis capacity. NCP or carrier DNA (control) was used in 5 fold molar excess to the Hrp3 enzymes. The reaction condition was 31 mM Na-Hepes, pH 7.8, 62 mM NaCl, 5 mM MgCl_2_, 0.1 mg/mL BSA, 5% Glycerol. The reaction was incubated for 15 min. at 30 °C to measure enzyme binding. The ATP mix, which contains 27 μL of 0.1 mM ATP and 2 uL Gamma ATP (6000 Ci/mmol) was added to a final volume of 29 μL, with the final concentration of ~8 μM and mixed for 15 min. The reaction was stopped by adding EDTA to a final concentration of 80 mM, and SDS with a final concentration of 1.6%. ATPase activity was monitored by performing Thin-Layer Chromatography (TLC). A sheet of PE cellulose was pre-run in sterile water and subsequently dried. A spot 1.0 μL of the stopped reaction was loaded to the prepared PE cellulose plate, making sure that all samples were spotted in the same line at the bottom. ATP and hydrolyzed Pi were separated by running the TLC in 0.5 M LiCl, 0.5 M Formic acid. The TLC plate was dried and exposed to a phosphor-imaging screen for about 10 min. Exposed images were quantified using the Quantity One software.

### 4.9. Western Blot Analysis

WT and domain deleted Hrp3 proteins were run on a 12% Tris-Bis gel. The gel was blotted on a PVDF membrane. The blot was incubated with primary antibodies anti-TAP (CBA1001, Thermo fisher, Waltham, MA, USA), anti-H3 (ab1791, Abcam, Cambridge, UK), and anti-Flag (M2, Merk, Kenilworth, NJ, USA). The secondary antibodies used were fluorescent IRDye^®^ 800CW Donkey anti-Rabbit IgG (red) and RDye^®^ 800CW Donkey anti-mouse IgG (green). The blots were developed in Odyssey^®^ CLx Imaging System from LI-COR Biosciences.

## 5. Conclusions

In this study, we have successfully established a protocol to purify *S. pombe* histones and reconstitute them into functional nucleosomes in vitro. This system will likely be useful for future studies of chromatin regulation in this popular model organism. We then used the reconstituted *S. pombe* nucleosomes to study the function of non-catalytic (non-ATPase) domains in the CHD1 homolog Hrp3 (chromo, SANT, SLIDE, and coupling region). We found that the association between Hrp3 and nucleosomes was reduced or abolished by all four domain deletions. Probably as a consequence of the reduced nucleosome affinity, the ATP hydrolyzing activities were strongly reduced in all four Hrp3 mutant constructs. Only the coupling region deletion showed some stimulation of ATPase activity by the addition of nucleosome substrate. The Hrp3 SANT deletion showed an increased preference for DNA as a substrate at higher enzyme concentrations suggesting that the SANT and SLIDE domains affect the DNA binding properties of Hrp3 in different ways. We conclude that SANT, SLIDE, and chromo-domains of Hrp3 contribute to its ATPase activity and nucleosome interactions in a non-redundant manner. Therefore, the functional role of each non-catalytic domain of Hrp3 in remodeling needs to be further addressed.

## Figures and Tables

**Table 1:** *S.pombe* strains used in this study.

Strain Name	Genotype	Source
Hu2402	*h- hrp1D::ura4(1/2 gene deleted), ade6-M210 leu1-32 ura4-D18 Hrp3_TAP*	This study
Hu3060	*h- hrp1D::ura4(1/2 gene deleted), ade6-M210 leu1-32 ura4-D18 Hrp3 chromodomain∆ _ TAP*	This study
Hu3061	*h- hrp1D::ura4(1/2 gene deleted), ade6-M210 leu1-32 ura4-D18 Hrp3 ∆sant _ TAP*	This study
Hu3062	*h- hrp1D::ura4(1/2 gene deleted), ade6-M210 leu1-32 ura4-D18 Hrp3 ∆slide _ TAP*	This study
Hu3063	*h- hrp1D::ura4(1/2 gene deleted), ade6-M210 leu1-32 ura4-D18 Hrp3 coupling region∆ _ TAP*	This study
Hu2204	*h- Hrp3::leu2+leu1-32 hrp1_2xFLAGKanMX*	This study

## Data Availability

Not applicable.

## Supplementary Materials

The following are available online at https://www.mdpi.com/1422-0067/22/4/1793/s1 Figure S1: Optimization of *S. pombe* histone H4 protein expression in different competent *E. coli* strains and spH3 and spH4 protein extractions from inclusion body, Figure S2: The domain deletion strategy used for Hrp3 in *S. pombe*., Figure S3. ATPase activities for wild type and different mutant Hrp3 proteins, Supplementary information: P199-1 plasmid sequences
