# Supplementary material for: The Role of Non-Catalytic Domains of Hrp3 in Nucleosome Remodeling
Source: Int J Mol Sci. 2021 Feb 11;22(4):1793. doi: 10.3390/ijms22041793 (PMC7918567; doi:10.3390/ijms22041793)
Supplement: Supplementary file 1 [file ijms-22-01793-s001.pdf]

Figure S1

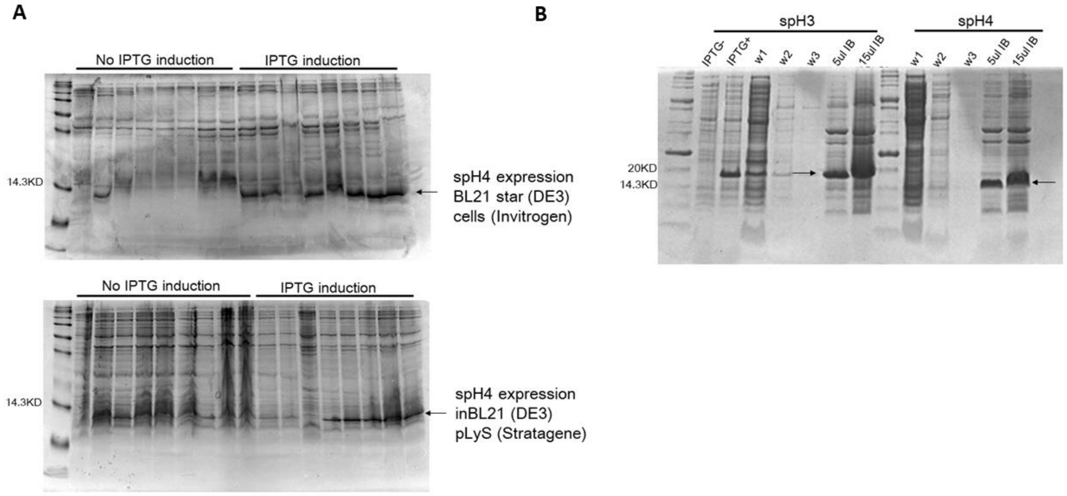

Figure S1: ***Optimization of S. pombe histone H4 protein expression in different competent E. coli strains and spH3/spH4 protein extractions from inclusion body*** *A) A small scale expression of spH4 in 5ml culture was set from different single colonies after transformation in BL21 star cells (upper panel) and BL21pLys cells (bottom-panel) with or without IPTG induction to identify the colony with highest levels of histone H4 expression. The crude cell lysate was denatured and protein was separated in 18% SDS-PAGE to identify the colony with high expression of spH4. BL21 star transformed cells showed higher spH4 protein expression than BL21 pLyS cells. B)* Histone proteins, *spH3 and spH4 were extracted from the inclusion bodies (IB), denatured, and separated in 18% SDS_PAGE. Mini-scale expression without (-) or with (+) IPTG induction, extraction after three washes (w1-3) were also loaded in the 18% SDS-PAGE, which showed that the histone proteins are retained in the inclusion bodies.*

Figure S2

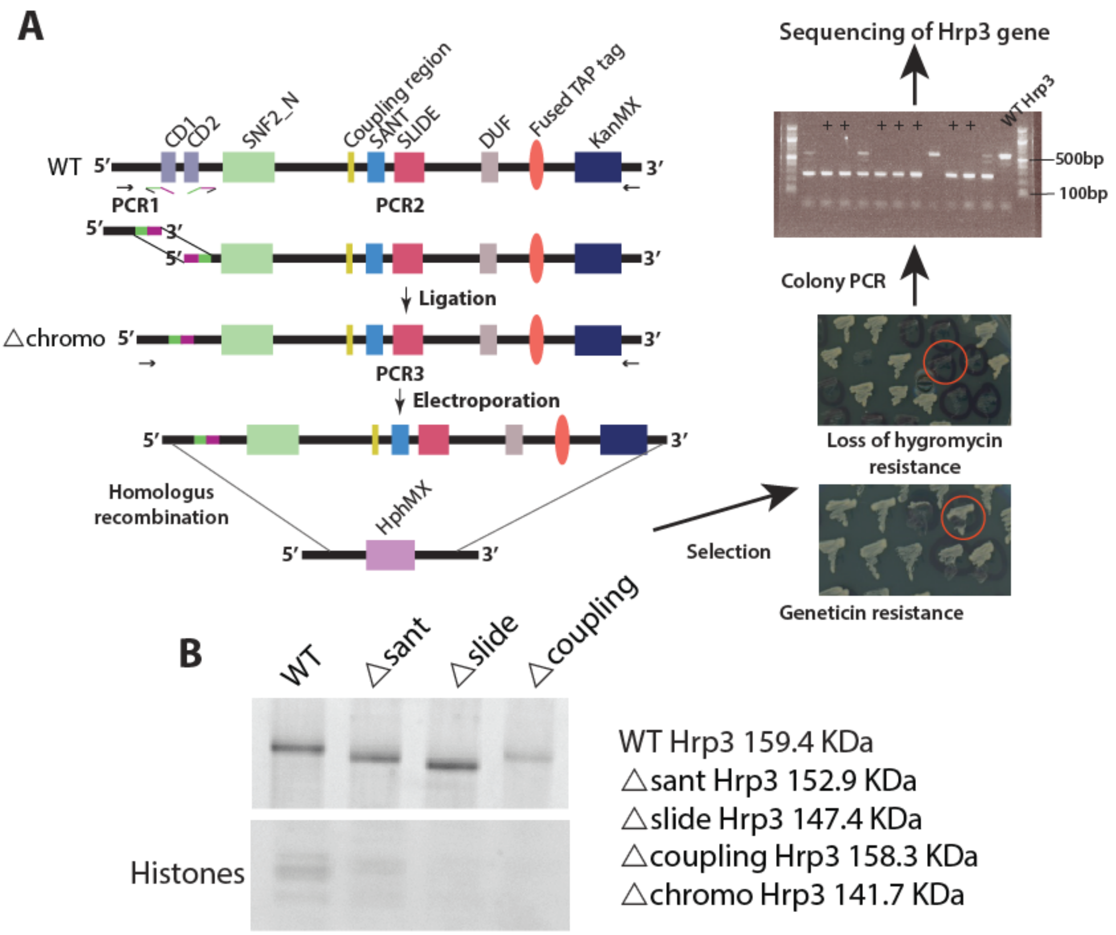

Figure S2: ***The domain deletion strategy used for Hrp3 in S. pombe.*** *A) Description of a representative chromo domain deletion strategy of Hrp3. Fragments from left and right segments of chromo-domains were amplified from the genomic DNA extracted from wild type Hrp3_TAP tagged strain at its C-terminus. The PCR fragments were ligated and transformed into S. pombe strains, where Δhrp3:HphMx. Homologous recombination at the hrp3 loci will switch HphMx cassette with KanMx cassette thereby switching the transformed strain from hygromycin resistance to kanamycin resistance. The KanMx resistance strains were then validated for domain deletion by PCR amplification and subsequently sequenced. This strategy was followed for all the internal domain deletion to generate mutant Hrp3 strains. B) The SDS-PAGE image of WT and mutant Hrp3 proteins (top panel) along with co-purified histones (bottom panel) and approximate molecular weight of proteins in KDa.*

Figure S3

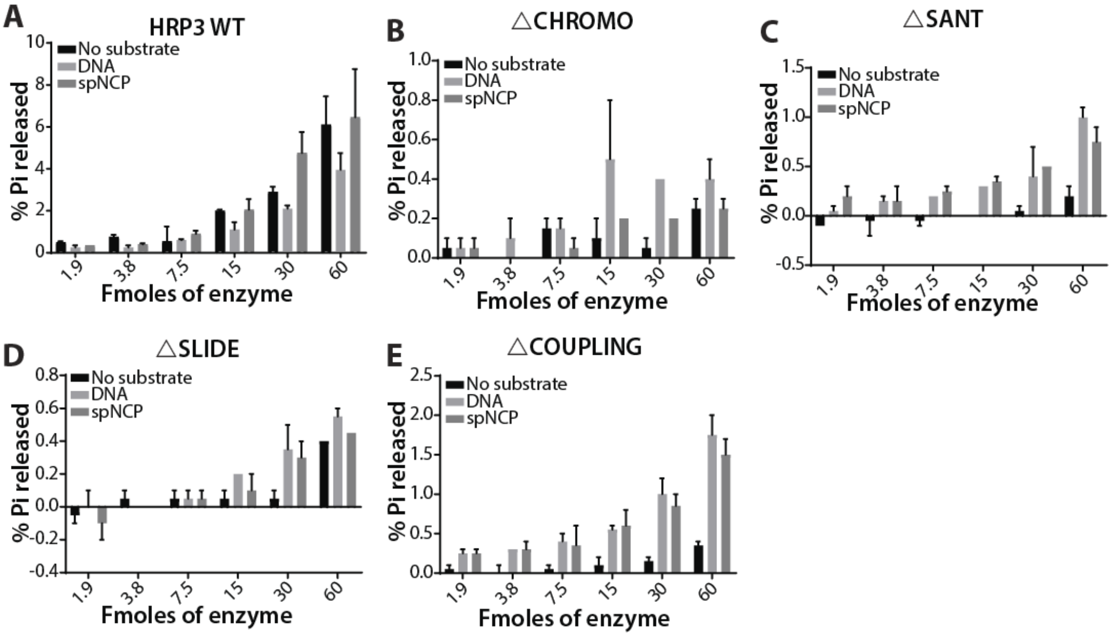

Figure S3. ***ATPase activities for wild type and different mutant Hrp3 proteins****: A mixture of γP*^{32}*-ATP and ATP were mixed with different amounts (femto moles) of wild type and mutant Hrp3 proteins (as indicated) in the presence of DNA and pombe nucleosomes along with no substrate control. Released isotope/free inorganic phosphate, Pi reflects the capacity of hydrolyzing ATP with DNA or sp70N0 nucleosome as substrates. No substrate (with only Hrp3) was used as a control. Data are represented as the mean value from two bio-replicate experiments. Error bar shows the standard error of the mean (SEM).*

Supplementary information:

P199-1 plasmid sequences + Nucleosome particle sequence

TCGCGCGTTTCGGTGATGACGGTGAAAACCTCTGACACATGCAGCTCCCGGAGACGGTCACAGCTTGTCTGTAAGCGGATG

CCGGGAGCAGACAAGCCCGTCAGGGCGCGTCAGCGGGTGTTGGCGGGTGTCGGGGCTGGCTTAACTATGCGGCATCAGAG

CAGATTGTACTGAGAGTGCACCATATGCGGTGTGAAATACCGCACAGATGCGTAAGGAGAAAATACCGCATCAGGCGCCATT

CGCCATTCAGGCTGCGCAACTGTTGGGAAGGGCGATCGGTGCGGGCCTCTTCGCTATTACGCCAGCTGGCGAAAGGGGGAT

GTGCTGCAAGGCGATTAAGTTGGGTAACGCCAGGGTTTTCCCAGTCACGACGTTGTAAAACGACGGCCAGTGAATTCGAGC

TCGGTACTCGGGAGCTCGGAACACTATCCGACTGGCACCGGCAAGGTCGCTGTTCAATACATGCACAGGATGTATATATCTG

ACACGTGCCTGGAGACTAGGGAGTAATCCCCTTGGCGGTTAAAACGCGGGGGACAGCGCGTACGTGCGTTTAAGCGGTGCT

AGAGCTTGCTACGACCAATTGAGCGGCCTCGGCACCGGGATTCTCTCGGGGATCCTCTAGAGTCGACCTGCAGGCATGCAA

GCTTGGCGTAATCATGGTCATAGCTGTTTCCTGTGTGAAATTGTTATCCGCTCACAATTCCACACAACATACGAGCCGGAAGC

ATAAAGTGTAAAGCCTGGGGTGCCTAATGAGTGAGCTAACTCACATTAATTGCGTTGCGCTCACTGCCCGCTTTCCAGTCGG

GAAACCTGTCGTGCCAGCTGCATTAATGAATCGGCCAACGCGCGGGGAGAGGCGGTTTGCGTATTGGGCGCTCTTCCGCTTC

CTCGCTCACTGACTCGCTGCGCTCGGTCGTTCGGCTGCGGCGAGCGGTATCAGCTCACTCAAAGGCGGTAATACGGTTATCC

ACAGAATCAGGGGATAACGCAGGAAAGAACATGTGAGCAAAAGGCCAGCAAAAGGCCAGGAACCGTAAAAAGGCCGCGT

TGCTGGCGTTTTTCCATAGGCTCCGCCCCCCTGACGAGCATCACAAAAATCGACGCTCAAGTCAGAGGTGGCGAAACCCGA

CAGGACTATAAAGATACCAGGCGTTTCCCCCTGGAAGCTCCCTCGTGCGCTCTCCTGTTCCGACCCTGCCGCTTACCGGATA

CCTGTCCGCCTTTCTCCCTTCGGGAAGCGTGGCGCTTTCTCATAGCTCACGCTGTAGGTATCTCAGTTCGGTGTAGGTCGTTC

GCTCCAAGCTGGGCTGTGTGCACGAACCCCCCGTTCAGCCCGACCGCTGCGCCTTATCCGGTAACTATCGTCTTGAGTCCAA

CCCGGTAAGACACGACTTATCGCCACTGGCAGCAGCCACTGGTAACAGGATTAGCAGAGCGAGGTATGTAGGCGGTGCTAC

AGAGTTCTTGAAGTGGTGGCCTAACTACGGCTACACTAGAAGGACAGTATTTGGTATCTGCGCTCTGCTGAAGCCAGTTACC

TTCGGAAAAAGAGTTGGTAGCTCTTGATCCGGCAAACAAACCACCGCTGGTAGCGGTGGTTTTTTTGTTTGCAAGCAGCAG

ATTACGCGCAGAAAAAAAGGATCTCAAGAAGATCCTTTGATCTTTTCTACGGGGTCTGACGCTCAGTGGAACGAAAACTCA

CGTTAAGGGATTTTGGTCATGAGATTATCAAAAAGGATCTTCACCTAGATCCTTTTAAATTAAAAATGAAGTTTTAAATCAATC

TAAAGTATATATGAGTAAACTTGGTCTGACAGTTACCAATGCTTAATCAGTGAGGCACCTATCTCAGCGATCTGTCTATTTCGT

TCATCCATAGTTGCCTGACTCCCCGTCGTGTAGATAACTACGATACGGGAGGGCTTACCATCTGGCCCCAGTGCTGCAATGAT

ACCGCGAGACCCACGCTCACCGGCTCCAGATTTATCAGCAATAAACCAGCCAGCCGGAAGGGCCGAGCGCAGAAGTGGTC

CTGCAACTTTATCCGCCTCCATCCAGTCTATTAATTGTTGCCGGGAAGCTAGAGTAAGTAGTTCGCCAGTTAATAGTTTGCGC

AACGTTGTTGCCATTGCTACAGGCATCGTGGTGTCACGCTCGTCGTTTGGTATGGCTTCATTCAGCTCCGGTTCCCAACGATC

AAGGCGAGTTACATGATCCCCCATGTTGTGCAAAAAAGCGGTTAGCTCCTTCGGTCCTCCGATCGTTGTCAGAAGTAAGTTG

GCCGCAGTGTTATCACTCATGGTTATGGCAGCACTGCATAATTCTCTTACTGTCATGCCATCCGTAAGATGCTTTTCTGTGACT

GGTGAGTACTCAACCAAGTCATTCTGAGAATAGTGTATGCGGCGACCGAGTTGCTCTTGCCCGGCGTCAATACGGGATAATA

CCGCGCCACATAGCAGAACTTTAAAAGTGCTCATCATTGGAAAACGTTCTTCGGGGCGAAAACTCTCAAGGATCTTACCGCT

GTTGAGATCCAGTTCGATGTAACCCACTCGTGCACCCAACTGATCTTCAGCATCTTTTACTTTCACCAGCGTTTCTGGGTGAG

CAAAAACAGGAAGGCAAAATGCCGCAAAAAAGGGAATAAGGGCGACACGGAAATGTTGAATACTCATACTCTTCCTTTTTC

AATATTATTGAAGCATTTATCAGGGTTATTGTCTCATGAGCGGATACATATTTGAATGTATTTAGAAAAATAAACAAATAGGGG

TTCCGCGCACATTTCCCCGAAAAGTGCCACCTGACGTCTAAGAAACCATTATTATCATGACATTAACCTATAAAAATAGGCGT

ATCACGAGGCCCTTTCGTCACGACGTTGT

S.pombe histone sequences cloned in the plasmids

> Htb1-2-1

CTGGGGCGGTACATTCCCCTCTAGAAATAATTTTGTTTAACTTTAAGAAGGAGATATACCATGTCTGCTGCTGAAAAGAAACC

CGCTTCCAAGGCTCCCGCCGGTAAGGCTCCTAGGGATACCATGAAGTCTGCTGATAAGAAGCGTGGTAAAAACAGAAAGGA

AACTTATTCATCCTATATTTACAAGGTGTTGAAGCAAGTTCACCCTGATACTGGTATTTCCAACCAAGCCATGCGTATCTTGAA

CTCTTTCGTCAACGATATTTTTGAGCGTATTGCCACTGAGGCTTCCAAGCTTGCTGCTTACAACAAGAAGTCCACTATTTCTT

CCCGTGAAATCCAGACTGCTGTTCGTTTGATTCTTCCCGGTGAGTTAGCCAAGCACGCCGTTACCGAAGGTACCAAATCCGT

CACCAAGTATTCTTCTTCTGCTCAGTAACTCGAGCACCACCACCACCACCACTGAGATCCGGCTGCTAACAAAGCCCGAAA

GGAAGCTGAGTTGGCTGCTGCCACCGCTGAGCAATAACTAGCATAACCCCTTGGGGCCTCTAAACGGGTCTTGAGGGGTTTT

TTGCTGAAAGGAGGAACTATATCCGGATTGGCGAATGGGACGCGCCCTGTAGCGGCGCATTAAGCGCGGCGGGTGTGGTGG

TTACGCGCAGCGTGACCGCTACACTTGCCAGCGCCCTAGCGCCCGCTCCTTTCGCTTTCTTCCCTTCCTTTCTCGCCACGTTC

GCCGGCTTTCCCCGTCAAGCTCTAAATCGGGGGCTCCCTTTAGGGTTCCGATTTAGTGCTTTACGGCACCTCGACCCCAAAA

AACTTGATTAGGGTGATGGTTCACGTAGTGGGCCATCGCCCTGATAGACGGTTTTTCGCCCTTTGACGTTGGAGTCCACGTTC

TTTAATAGTGGACTCTTGTTCCAAACTGGAACAACACTCAACCCTATCTCGGTCTATTCTTTTGATTTATAAGGATTTTGCCGA

TTTCGGCCTATTGGTTAAAAAATGAGCTGATTTAACAAAATTTAACGCGATTTAACAAATATTAACGCTTACAATTTAGTGGCAC

TTTTCGGGAAATGTGCGCGACCCCTATTTGTTTATTTTTCTAATACATTCAATATGTATCCGCTCATGATATCTAGAAAACTCAT

CGAGCATCAATGAACTGCATTATCATATCAGATATCATACATATCTTTGAAAAGCGTTTCGTTATGAGAAACTCACGAGGCGTT

CCATAGATGGCGATCGGATCGCTCGATTCCGACTGCCCCAACTATAACCCTTAATTCTCCTGCGCGGAGTAG

>hhf1-cloning

GGGGTGGGGACGGGTACATTCCCCTCTAGAATAATTTTGTTTAACTTTAAGAAGGAGATATACCATGTCTGGTCGTGGTAAAG

GTGGTAAAGGTTTGGGAAAAGGTGGTGCTAAGCGTCACCGTAAAATTCTTCGTGACAACATTCAAGGTATTACTAAGCCTGC

TATCCGTCGTCTTGCTCGTCGTGGTGGTGTGAAGCGTATTTCCGCTTTGGTTTATGAAGAGACTCGTGCTGTTCTCAAGCTCT

TCTTAGAAAATGTTATTCGCGATGCCGTCACCTATACTGAGCATGCCAAGCGTAAGACTGTCACTTCTTTGGACGTTGTCTATT

CTTTGAAGCGTCAAGGCCGTACCATTTATGGTTTCGGTGGTTAACTCGAGCACCACCACCACCACCACTGAGATCCGGCTGC

TAACAAAGCCCGAAAGGAAGCTGAGTTGGCTGCTGCCACCGCTGAGCAATAACTAGCATAACCCCTTGGGGCCTCTAAACG

GGTCTTGAGGGGTTTTTTGCTGAAAGGAGGAACTATATCCGGATTGGCGAATGGGACGCGCCCTGTAGCGGCGCATTAAGCG

CGGCGGGTGTGGTGGTTACGCGCAGCGTGACCGCTACACTTGCCAGCGCCCTAGCGCCCGCTCCTTTCGCTTTCTTCCCTTC

CTTTCTCGCCACGTTCGCCGGCTTTCCCCGTCAAGCTCTAAATCGGGGGCTCCCTTTAGGGTTCCGATTTAGTGCTTTACGGC

ACCTCGACCCCAAAAAACTTGATTAGGGTGATGGTTCACGTAGTGGGCCATCGCCCTGATAGACGGTTTTTCGCCCTTTGAC

GTTGGAGTCCACGTTCTTTAATAGTGGACTCTTGTTCCAAACTGGAACAACACTCAACCCTATCTCGGTCTATTCTTTTGATTT

ATAAGGGATTTTGCCGATTTCGGCCTATTGGTTAAAAATGAGCTGATTTAACAAAAATTTAACGCGAATTTTAACAAAATATTA

ACGCTTACAATTTAGGTGGCACTTTTCGGGGAAATGTGCGCGGAACCCCTATTTGTTTATTTTTCTAAATACATTCAATATGTAT

CCGCTCATGAATTATTCTTAGAAAACTCATCGAGCATCAAATGAAACTGCAATTTATTCATATCAGGATTATCAATACCATATTT

TTGAAAAGCCGTTCTGTAATGAAGGAGAAACTTCACCGGAG

>hta2-cloning

GTGGGTGGAACGGGACATTCCCCTCTAGAAATAATTTTGTTTAACTTTAAGAAGGAGATATACCATGTCTGGAGGTAAATCTG

GTGGTAAGGCCGCAGTCGCCAAGTCTGCTCAATCTCGTTCCGCTAAGGCTGGTTTGGCCTTCCCTGTCGGTCGTGTTCATCG

TTTGTTGCGTAAGGGTAATTATGCTCAACGTGTTGGTGCTGGTGCTCCCGTTTACTTGGCTGCCGTCTTGGAGTATTTAGCTG

CCGAAATCCTCGAATTGGCCGGTAATGCTGCTCGTGATAACAAGAAGACTCGTATCATTCCCCGTCATCTTCAATTGGCTATT

CGCAATGACGAAGAATTGAACAAACTACTTGGTCATGTTACTATTGCCCAGGGTGGTGTTGTACCTAATATCAACGCTCATCT

CTTGCCCAAACAATCTGGTAAGGGCAAGCCTAGCCAAGAGCTTTAACTCGAGCACCACCACCACCACCACTGAGATCCGGC

TGCTAACAAAGCCCGAAAGGAAGCTGAGTTGGCTGCTGCCACCGCTGAGCAATAACTAGCATAACCCCTTGGGGCCTCTAA
ACGGGTCTTGAGGGGTTTTTTGCTGAAAGGAGGAACTATATCCGGATTGGCGAATGGGACGCGCCCTGTAGCGGCGCATTAA
GCGCGGCGGGTGTGGTGGTTACGCGCAGCGTGACCGCTACACTTGCCAGCGCCCTAGCGCCCGCTCCTTTCGCTTTCTTCCC
TTCCTTTCTCGCCACGTTCGCCGGCTTTCCCCGTCAAGCTCTAAATCGGGGGCTCCCTTTAGGGTTCCGATTTAGTGCTTTAC
GGCACCTCGACCCCAAAAAACTTGATTAGGGTGATGGTTCACGTAGTGGGCCATCGCCCTGATAGACGGTTTTTCGCCCTTT
GACGTTGGAGTCCACGTTCTTTAATAGTGGACTCTTGTTCCAACTGGAACAACACTCAACCCTATCTCGGTCTATTCTTTTGA
TTATAAGGATTTTGCCGATTTCGGCCTATTGGTTAAAAAATGAGCTGATTTAACAAAAATTTAACGCGAATTTTAACAAAATATT
AACGCTTACAATTTAGGTGGCACTTTTCGGGGAAATGTGCGCGAACCCCTATTTGTTTATTTTTCTAAATACATTCAAATATGTATC
GCTCATGATAATTCTTAGAAAAACTCATCGAGCATCAATGAACTGCATTATTCATATCAGGATATCATTCTATTTTTGAAAAGCC
GTTTCTGTAATGAAGAGAAAAACTCTCCGAGGCGAG

> hht1

TAATTTTGTTTAACTTTAAGAAGGAGATATACCATGGCTCGTACTAAACAAACAGCTCGTAAGTCTACCGGTGGTAAGGCACC
CCGTAAGCAATTGGCCTCTAAGGCCGCTCGTAAGGCCGCTCCCGCTACCGGAGGTGTTAAGAAGCCTCATCGTTATCGTCCT
GGTACTGTCGCTCTTCGTGAGATTCGTCGTTATCAAAAGTCTACTGAACTTTTAATTCGTAAGCTACCTTTCCAACGTTTGGTC
CGTGAAATCGCCCAAGATTTCAAGACTGACTTGCGTTTCCAATCTTCTGCCATTGGTGCTCTCCAAGAAGCTGTTGAGGCCT
ACCTTGTCTCTCTATTTGAGGACACTAACTTGTGTGCTATTCACGGAAAACGTGTTACGATTCAACCCAAGGATATGCAGTTG
GCTCGTCGTCTCCGTGGCGAACGCTCATAACTCGAGCACCACCACCACCACCACTGAGATCCGGCTGCTAACAAAGCCCGA
AAGGAAGCTGAGTTGGCTGCTGCCACCGCTGAGCAATAACTAGCATAACCCCTTGGGGCCTCTAAACGGGTCTTGAGGGGT
TTTTTGCTGAAAGGAGGAACTATATCCGGATTGGCGAATGGGACGCGCCCTGTAGCGGCGCATTAAGCGCGGCGGGTGTGGT
GGTTACGCGCAGCGTGACCGCTACACTTGCCAGCGCCCTAGCGCCCGCTCCTTTCGCTTTCTTCCCTTCCTTTCTCGCCACGT
TCGCCGGCTTTCCCCGTCAAGCTCTAAATCGGGGGCTCCCTTTAGGGTTCCGATTTAGTGCTTTACGGCACCTCGACCCCAA
AAAACTTGATTAGGGTGATGGTTCACGTAGTGGGCCATCGCCCTGATAGACGGTTTTTCGCCCTTTGACGTTGGAGTCCACG
TTCTTTAATAGTGGACTCTTGTTCCAAACTGGAACAACACTCAACCCTATCTCGGTCTATTCTTTTGATTTATAAGG
